# Regional Differences in Hospital Costs of Acute Ischemic Stroke in China: Analysis of Data From the Chinese Acute Ischemic Stroke Treatment Outcome Registry

**DOI:** 10.3389/fpubh.2021.783242

**Published:** 2021-12-10

**Authors:** Yuxuan Lu, Weiping Sun, Zhiyuan Shen, Wei Sun, Ran Liu, Fan Li, Junlong Shu, Liwen Tai, Guozhong Li, Huisheng Chen, Guiru Zhang, Lei Zhang, Xuwen Sun, Jinhua Qiu, Yan Wei, Haiqiang Jin, Yining Huang

**Affiliations:** ^1^Department of Neurology, Peking University First Hospital, Beijing, China; ^2^Department of Neurology, Second Hospital of Hebei Medical University, Shijiazhuang, China; ^3^Department of Neurology, First Affiliated Hospital of Harbin Medical University, Neurology, Harbin, China; ^4^Department of Neurology, The General Hospital of Shenyang Military Command, Shenyang, China; ^5^Department of Neurology, Penglai People's Hospital, Penglai, China; ^6^Department of Neurology, Fifth Affiliated Hospital of Sun Yat-sen University, Zhuhai, China; ^7^Department of Neurology, Qingdao University Medical College Affiliated Yantai Yuhuangding Hospital, Yantai, China; ^8^Department of Neurology, Huizhou First Hospital, Huizhou, China; ^9^Department of Neurology, Harrison International Peace Hospital, Hengshui, China

**Keywords:** hospital costs, ischemic stroke, regional differences, China, determinants

## Abstract

**Background and Purpose:** Studies on the regional differences in hospital costs of acute ischemic stroke (AIS) are scarce in China. We aimed to explore the regional differences in hospital costs and identify the determinants of hospital costs in each region.

**Methods:** Data were collected from the Chinese Acute Ischemic Stroke Treatment Outcome Registry (CASTOR), a multicenter prospective study on patients diagnosed with AIS and hospitalized from 2015 to 2017. Univariate and multivariate analyses were undertaken to identify the determinants of hospital costs of AIS.

**Results:** A total of 8,547 patients were included in the study, of whom 3,700 were from the eastern area, 2,534 were from the northeastern area, 1,819 were from the central area, and 494 were from the western area. The median hospital costs presented a significant difference among each region, which were 2175.9, 2175.1, 2477.7, and 2282.4 dollars in each area, respectively. Each region showed a similar hospital cost proportion size order of cost components, which was Western medicine costs, other costs, diagnostic costs, and traditional medicine costs, in descending order. Male sex, diabetes mellitus, severe stroke symptoms, longer length of stay, admission to the intensive care unit, in-hospital complications of hemorrhage, and thrombectomy were independently associated with hospital costs in most regions.

**Conclusion:** Hospital costs in different regions showed a similar proportion size order of components in China. Each region had different determinants of hospital costs, which reflected its current medical conditions and provided potential determinants for increasing medical efficiency according to each region's situation.

## Introduction

As the second leading cause of death and third leading cause of disability globally, stroke places an enormous burden on current health care and social costs (1). Ischemic stroke (IS) is the most common subtype, accounting for 69.6–79.1% of all stroke cases with a crude hospital mortality of 0.88% in China, which raises an increasingly serious issue regarding national health care costs (2, 3). Several studies have been conducted to identify the determinants of hospital costs of acute ischemic stroke (AIS) in mainland China (4–12). However, no study has sought to identify the regional differences in hospital costs considering the huge geographic and socioeconomic differences among regions in China. In this study, we aimed to explore regional differences in hospital costs of AIS and identify each region's potential determinants for increasing medical efficiency in China.

## Methods

### Study Design

Data were obtained from the Chinese Acute Ischemic Stroke Treatment Outcome Registry (CASTOR), a multicenter prospective study that included 10,002 cases of AIS from 80 hospitals in 44 cities across mainland China. The trial design and protocol have been described previously (13). The hospitals included in the study were required to have a neurology ward with over 100 stroke patients admitted each year. The inclusion criteria for patients were (1) age ≥18 years; (2) diagnosis of AIS confirmed by computed tomography (CT) or magnetic resonance imaging (MRI) from May 2015 to October 2017; and (3) admission to a hospital within 7 days of onset of AIS. Patients with hemorrhagic stroke were excluded. All treatments would be selected by the investigators according to the symptoms and medical history of patients as recommended by the Chinese Guidelines for the Diagnosis and Treatment of Acute Ischemic Stroke (14).

### Explanatory Variables

**Figure 1:**
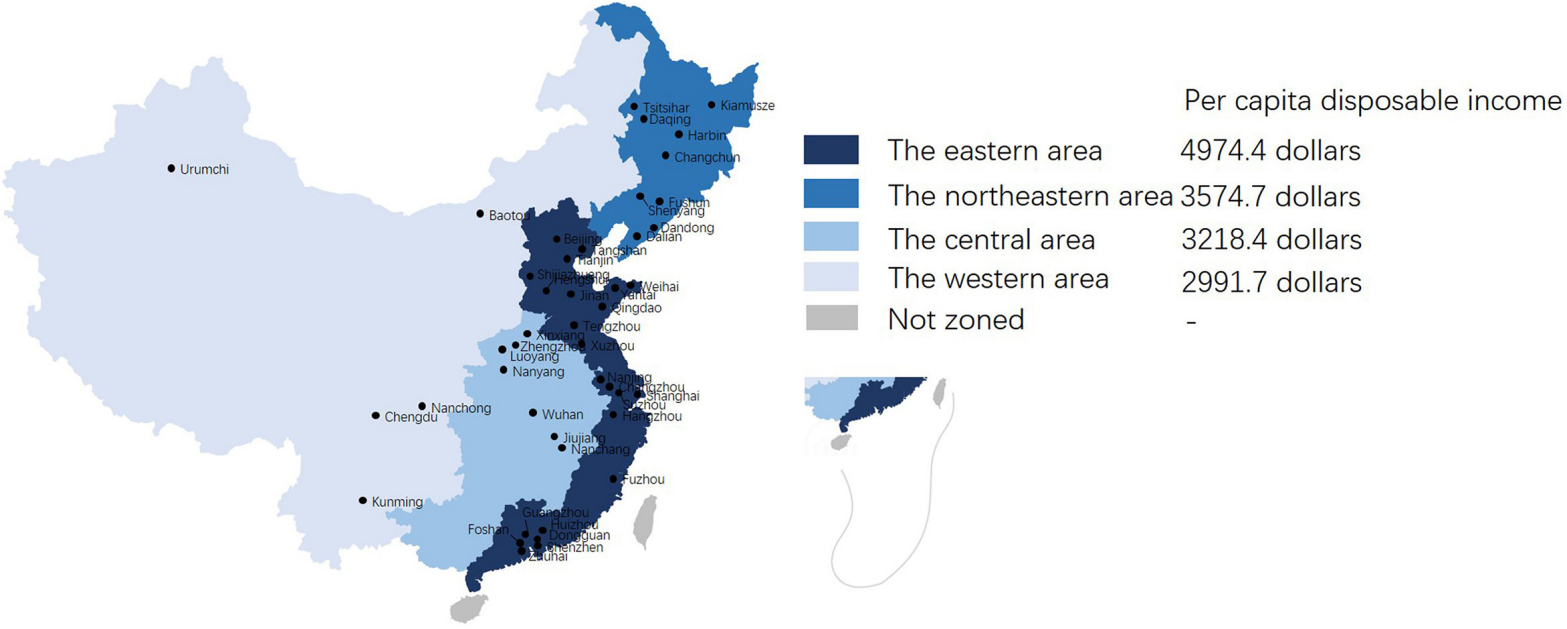
Study site locations and division of geographical regions.

The covariates included demographic variables, medical history, and clinical features of the stroke index, including age and sex; payment type; medical history of prior stroke (including ischemic stroke and hemorrhagic stroke), hypertension, diabetes mellitus (DM), coronary heart disease (CHD: any history of heart attack and/or myocardial infarction, angina, or coronary heart disease), or atrial fibrillation (AF); geographic region; hospital level; National Institutes of Health Stroke Scale (NIHSS) score on admission (mild: 0–5; moderate: 6–10; severe: >10); length of stay (LOS); use of the intensive care unit (ICU); in-hospital complications including infection, deep venous thrombosis (DVT), cardiac events, hemorrhage, and serious falls; use of thrombolysis; and thrombectomy. Hypertension was defined as a history of elevated blood pressure >140/90 mmHg on two independent occasions or the use of antihypertensive medication. DM was defined as a history of elevated blood glucose on 2 independent occasions or the use of antidiabetic medication. AF was defined as a history of AF or characteristics of AF identified by ECG during hospitalization. The participating hospitals were divided into four groups according to their geographical region: the eastern area, the northeastern area, the central area, and the western area (15). Data from the National Bureau of Statistics showed that per capita disposable income in the different regions was 4974.4, 3574.7, 3218.4, and 2991.7 dollars, respectively, in 2017. Figure 1 shows the study site location and division of geographical regions. Hospital level was divided into two groups according to whether the participating hospitals were tertiary hospitals (large hospitals serving as major tertiary referral centers in the provincial capitals and major cities with >1,000 beds, all located in the center of the city). Besides tertiary hospitals, the remaining were secondary hospitals (regional hospitals operating in an authority administration area with at least 100 inpatient beds providing acute medical care and preventive care services to populations of at least 100,000 people) (15). The definition of in-hospital complications was in accordance with previous studies (16, 17). Infection included urinary tract infection, chest infection, and other infection. Hemorrhage included gastrointestinal hemorrhage, urinary tract hemorrhage, dermal ecchymosis, and hemorrhagic transformation of ischemic stroke (18).

Hospital costs were divided into four parts: diagnostic costs, Western medicine costs, traditional medicine costs, and other costs. Diagnostic costs included the laboratory and examination costs for pathological examinations, laboratory tests, and imaging examinations. Western medicine costs included the costs of various Western medicines. Traditional medicine costs included the costs of traditional herbs, traditional patent medicine, and traditional operation treatment such as acupuncture and Chinese massage. Other costs mainly included labor costs, such as medical service costs, nursing costs, and surgery costs. A mean exchange rate of US $ 1 = 6.75 CNY was used for 2017. The functional outcome was evaluated with the modified Rankin Scale (mRS) at discharge (poor outcome: mRS > 2) (13).

### Statistical Analysis

Continuous variables were presented as mean ± standard error or median [interquartile range (IQR)] and were analyzed with Student's *t*-test or Wilcoxon rank-sum test as appropriate according to whether they were normally distributed. Categorical variables were presented as frequencies (percentages) and were analyzed with the chi-squared test. A multivariate analysis was used with multiple linear regression models with the “backward” method, and the levels of significance were a *P* < 0.05 for inclusion and a *P* > 0.1 for exclusion in the stepwise procedure, which included all covariates mentioned above (demographic variables, medical history, and clinical features of the index stroke) (11). Subgroup analysis was conducted to examine the regional differences in total hospital costs by grouping patients with different stroke severities and with/without thrombolysis. Multivariate analysis could not be performed to investigate the regional differences in traditional medicine costs due to the low cost and low prevalence of traditional medicine use. A *P*-value of 0.05 (two tailed) was considered indicative of significance. All statistical analyses were performed with the IBM SPSS 22.0 software package (IBM, Armonk, NY, USA).

## Results

Of the 10,002 patients initially screened for eligibility, 1,455 patients were excluded, including patients who dropped out of the study (*n* = 48) and patients with missing data (*n* = 1,407). A total of 8,547 patients were included in the final analysis. The median age of the overall cohort was 64.0 years (IQR: 56.0–73.0), and 5,604 (65.6%) participants were male. 1,497 (17.5%) patients paid at their own expense, and 7,050 (82.5%) patients had medical insurance, in which 154 (1.8%) patients had private insurance and 6,896 (80.7%) patients had public insurance. The median hospital cost was 2248.0 dollars (IQR: 1507.3–3339.2). Five (0.1%) patients died at discharge in this study. Patients from areas with higher per capita disposable income, such as the eastern area and northeastern area, tended to have DM, AF, milder stroke severity, and shorter LOS. Regarding hospital costs, patients from areas with higher per capita disposable income tended to have lower total hospital costs, lower Western medicine costs, and lower other costs with higher diagnostic costs (Table 1).

**Table 1:** Basic characteristics of different regions.

**Variables**	**The eastern area**	**The northeastern area**	**The central area**	**The western area**	***P*-value**
Male, *n* (%)	2,439 (65.9%)	1,684 (66.5%)	1170 (64.3%)	311 (63.0%)	0.280
Age, year, median (IQR)	65.0 (56.0–74.0)	63.0 (56.0–71.0)	64.0 (56.0–73.0)	65.0 (54.8–74.0)	<0.001
Medical insurance, *n* (%)	2,929 (79.2%)	2,117 (83.5%)	1,649 (90.7%)	355 (71.9%)	<0.001
**Medical history**					
Stroke, *n* (%)	690 (18.6%)	810 (32.0%)	447 (24.6%)	97 (19.6%)	<0.001
Hypertension, *n* (%)	2,412 (65.2%)	1,625 (64.1%)	1,187 (65.3%)	273 (55.3%)	<0.001
DM, *n* (%)	961 (26.0%)	677 (26.7%)	423 (23.3%)	106 (21.5%)	0.010
CHD, *n* (%)	460 (12.4%)	425 (16.8%)	251 (13.8%)	46 (9.3%)	<0.001
AF, *n* (%)	163 (4.4%)	141 (5.6%)	58 (3.2%)	19 (3.8%)	0.002
Tertiary hospital, *n* (%)	3,074 (83.1%)	2,426 (95.7%)	1,819 (100%)	494 (100%)	<0.001
**Stroke severity**					<0.001
Mild (NIHSS 0–5), *n* (%)	2,339 (63.2%)	1702 (67.2%)	1,107 (60.9%)	296 (59.9%)	
Moderate (NIHSS 6–10), *n* (%)	851 (23.0%)	515 (20.3%)	453 (24.9%)	115 (23.3%)	
Severe (NIHSS > 10), *n* (%)	510 (13.8%)	317 (12.5%)	259 (14.2%)	83 (16.8%)	
Length of stay, day, median (IQR)	12.0 (9.0–15.0)	10.0 (7.0–13.0)	14.0 (11.0–18.0)	14.0 (11.0–19.0)	<0.001
ICU, *n* (%)	44 (1.2%)	210 (8.3%)	65 (3.6%)	29 (5.9%)	<0.001
**In-hospital complications**					
Infection, *n* (%)	216 (5.8%)	260 (10.3%)	109 (6.0%)	20 (4.0%)	<0.001
DVT, *n* (%)	3 (0.1%)	5 (0.2%)	0 (0%)	1 (0.2%)	0.158
Cardiac events, *n* (%)	10 (0.3%)	11 (0.4%)	4 (0.2%)	0 (0%)	0.405
Hemorrhage, *n* (%)	70 (1.9%)	50 (2.0%)	36 (2.0%)	8 (1.6%)	0.956
Serious falls, *n* (%)	7 (0.2%)	8 (0.3%)	3 (0.2%)	0 (0%)	0.594
Thrombolysis, *n* (%)	125 (3.4%)	203 (8.0%)	51 (2.8%)	15 (3.0%)	<0.001
Thrombectomy, *n* (%)	2 (0.1%)	6 (0.2%)	7 (0.4%)	8 (1.6%)	<0.001
**Hospitalization costs, in dollars**					
Total costs, median (IQR)	2175.9 (1480.3–3331.4)	2175.1 (1446.1–3250.6)	2477.7 (1655.7–3496.2)	2282.4 (1696.7–3107.7)	<0.001
Diagnostic costs, median (IQR)	321.2 (219.5–456.3)	326.8 (165.6–488.9)	256.4 (51.0–408.1)	277.6 (195.3–468.6)	<0.001
Western medicine costs, median (IQR)	977.0 (569.2–1686.0)	1015.3 (615.4–1598.0)	1323.9 (758.8–1996.3)	1063.9 (705.5–1536.4)	<0.001
Traditional medicine costs, median (IQR)	7.3 (0.0–158.9)	21.4 (0–261.1)	111.6 (16.9–253.3)	0.0 (0.0–3.1)	<0.001
Other costs, median (IQR)	640.4 (400.4–1045.9)	524.4 (360.0–1040.0)	614.7 (389.6–1044.8)	782.3 (549.5–1294.1)	<0.001
Poor outcome (mRS > 2)	1,168 (31.6%)	726 (28.7%)	427 (23.5%)	173 (35.0%)	<0.001

*IQR, interquartile range; DM, diabetes mellitus; CHD, coronary heart disease; AF, atrial fibrillation; NIHSS, the National Institutes of Health Stroke Scale; ICU, intensive care unit; DVT, deep venous thrombosis; mRS, the modified Rankin Scale*.

**Figure 2:**
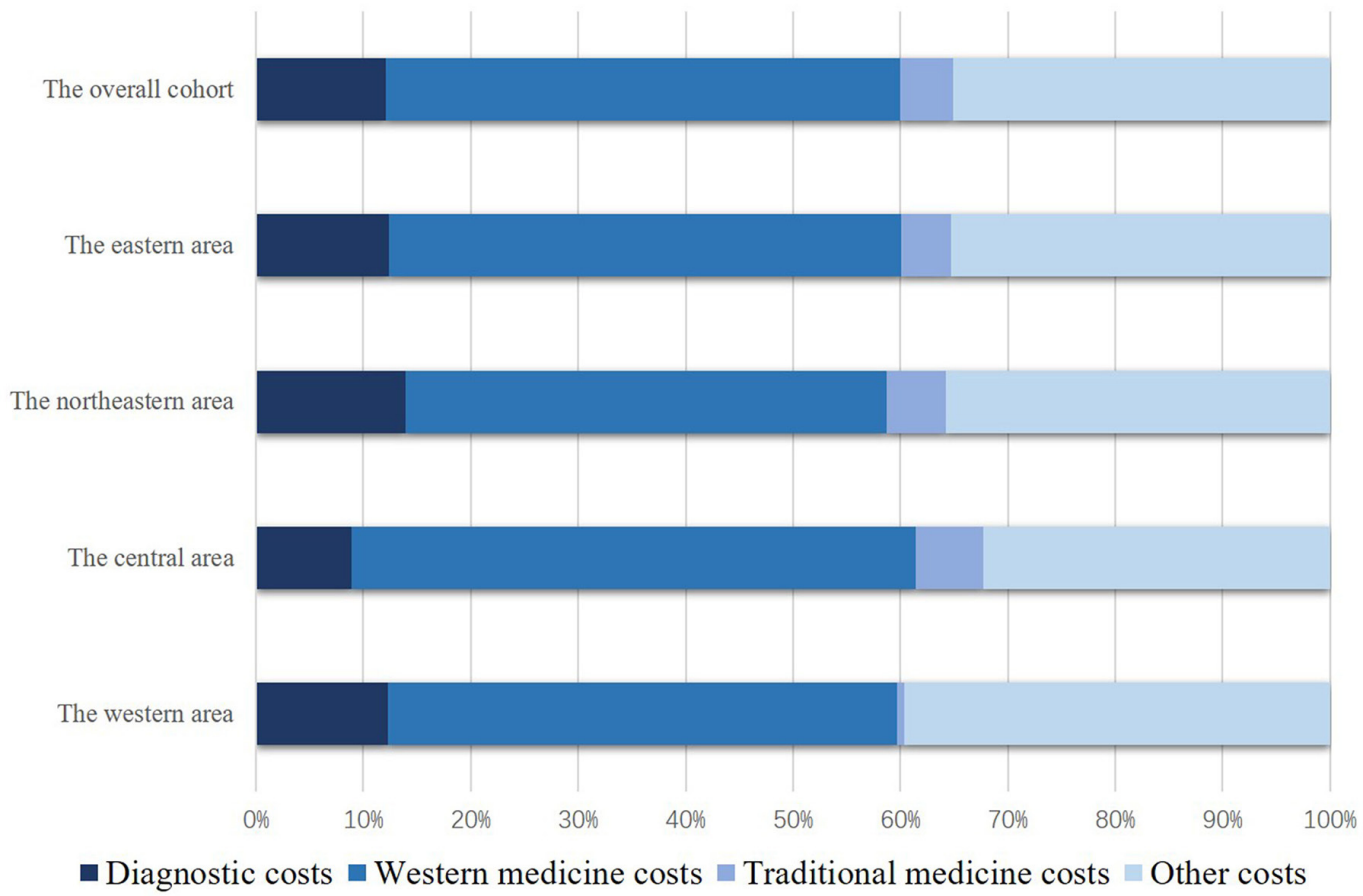
Cost components of the different regions.

Components of hospital costs also showed significant differences among different regions, while most of the hospital costs in different regions were Western medicine costs. All areas showed a similar proportion size order of cost components, which was Western medicine costs, other costs, diagnostic costs, and traditional medicine costs, in descending order (Figure 2). Multivariate analysis suggested that patients from areas with higher per capita disposable income tended to have higher total hospital costs, subgroup analysis showed similar results of regional differences on total hospital costs. Patients from the central area tended to have lower diagnostic costs and higher Western medicine costs (Tables 2–4).

**Table 2:** Association between regions and each component of hospital costs.

		**The central area**	**The eastern area**	**The northeastern area**	**The western area**
Total hospital costs	Coefficient (95% CI)	Reference	332.4 (217.1, 447.7)	163.1 (40.7, 285.5)	−117.8 (−314.4, 78.8)
	*P*-value	Reference	<0.001	0.009	0.240
Diagnostic costs	Coefficient (95% CI)	Reference	112.4 (97.6, 127.2)	128.3 (112.6, 144.0)	696.3 (71.1, 121.5)
	*P*-value	Reference	<0.001	<0.001	<0.001
Western medicine costs	Coefficient (95% CI)	Reference	8.2 (−50.0, 66.4)	−161.9 (−223.7, −100.1)	−170.9 (−270.1, −71.6)
	*P*-value	Reference	0.782	<0.001	0.001
Other costs	Coefficient (95% CI)	Reference	244.0 (170.1, 317.8)	202.3 (124.1, 280.6)	125.9 (0, 251.8)
	*P*-value	Reference	<0.001	<0.001	0.050

*CI, confidence interval*.

**Table 3:** Association between regions and total hospital costs in different stroke severity groups.

		**The central area**	**The eastern area**	**The northeastern area**	**The western area**
Mild group (NIHSS 0–5)	Coefficient (95% CI)	Reference	111.2 (−6.8, 229.3)	100.2 (−21.8, 222.2)	−229.2 (−431.1, −27.4)
	*P*-value	Reference	0.065	0.107	0.026
Moderate group (NIHSS 6–10)	Coefficient (95% CI)	Reference	763.1 (522.9, 1003.4)	537.9 (265.8, 810.0)	3.6 (−417.0, 424.2)
	*P*-value	Reference	<0.001	<0.001	0.987
Severe group (NIHSS > 10)	Coefficient (95% CI)	Reference	562.9 (96.7, 1029.2)	−199.4 (−725.9, 327.1)	−365.3 (−1121.4, 390.7)
	*P*-value	Reference	0.018	0.458	0.343

*NIHSS, the National Institutes of Health Stroke Scale; CI, confidence interval*.

**Table 4:** Association between regions and total hospital costs in groups with or without thrombolysis.

		**The central area**	**The eastern area**	**The northeastern area**	**The western area**
Group with thrombolysis	Coefficient (95% CI)	Reference	1138.7 (287.3, 1990.1)	1314.9 (520.7, 2109.0)	−922.6 (−2354.2, 508.9)
	*P*-value	Reference	0.009	0.001	0.206
Group without thrombolysis	Coefficient (95% CI)	Reference	302.1 (187.5, 416.8)	109.0 (−13.5, 231.5)	−110.4 (−306.1, 85.2)
	*P*-value	Reference	<0.001	0.081	0.269

*NIHSS, the National Institutes of Health Stroke Scale; CI, confidence interval*.

In the eastern area, patients with high hospital costs tended to be male and younger and have hypertension, DM, AF, more severe stroke symptoms, longer LOS, admission to the ICU, in-hospital complications of infection and hemorrhage, and thrombolysis. They tended to be from tertiary hospitals and did not have a history of stroke. In the northeastern area, patients with high hospital costs were male, younger, and from tertiary hospitals and had medical insurance; DM; more severe stroke symptoms; longer LOS; admission to the ICU; in-hospital complications of infection, cardiac events, and hemorrhage; thrombolysis; and thrombectomy. Patients with a history of CHD tended to have low hospital costs. In the central area, patients with high hospital costs tended to be male and have DM, AF, more severe stroke symptoms, longer LOS, admission to the ICU, in-hospital complications of hemorrhage, and thrombectomy. Patients with a history of stroke tended to have low hospital costs. In the western area, patients with high hospital costs tended to have hypertension, more severe stroke symptoms, longer LOS, admission to the ICU, in-hospital complications of hemorrhage, and thrombectomy (Table 5).

**Table 5:** Multivariate analysis of hospital costs in different areas.

**Variables**	**The eastern area**		**The northeastern area**		**The central area**		**The western area**	
	**Coefficient (95% CI)**	***P*-value**	**Coefficient (95% CI)**	***P*-value**	**Coefficient (95% CI)**	***P*-value**	**Coefficient (95% CI)**	***P*-value**
Male	301.6 (161.5, 441.7)	<0.001	264.7 (104.4, 424.9)	0.001	265.2(91.6, 438.8)	0.003	–	–
Age, year	−8.0 (−13.5, −2.6)	0.004	−7.3 (−14.2, −0.4)	0.040	–	–	–	–
Medical insurance	–	–	235.0 (30.1, 440.0)	0.025	–	–	−322.8 (−654.4, 8.7)	0.056
**Medical history**								
Stroke	−249.3(−418.0, −80.5)	0.004	−148.3(−309.9, 13.3)	0.072	−319.8(−512.2, −127.4)	0.001	–	–
Hypertension	238.7(99.4, 378.0)	0.001	–	–	–	–	413.6 (115.7, 711.4)	0.007
DM	174.4(24.9, 323.9)	0.022	264.0 (95.2, 432.9)	0.002	219.1(22.9, 415.2)	0.029	345.2 (−16.3, 706.6)	0.061
CHD	–	–	−308.8 (−512.8, −104.8)	0.003	–	–	–	–
AF	356.7(33.9, 679.5)	0.030	–	–	1151.2(678.8,1623.5)	<0.001	–	–
Tertiary hospital	748.8(573.1, 924.4)	<0.001	1608.7(1232.6, 1984.7)	<0.001	–	–	–	–
Stroke severity	710.6(617.2, 804.0)	<0.001	245.0(132.9, 357.1)	<0.001	263.8(145.5, 382.1)	<0.001	390.8(183.4, 598.1)	<0.001
Length of stay, day	59.0 (52.8, 65.3)	<0.001	146.9 (130.3, 163.6)	<0.001	138.7 (127.4, 150.0)	<0.001	108.5 (95.0, 121.9)	<0.001
ICU	3028.4(2425.0, 3631.9)	<0.001	3068.3(2766.5, 3370.1)	<0.001	1834.3(1382.2, 2286.4)	<0.001	968.5(343.8, 1593.3)	0.002
**In-hospital complications**								
Infection	912.3 (627.6, 1197.0)	<0.001	1085.7 (831.7, 1339.6)	<0.001	–	–	–	–
Cardiac events	–	–	1283.6(152.5–2414.8)	0.026	–	–	–	–
Hemorrhage	712.0 (229.2, 1194.9)	<0.001	1778.3(1235.7, 2321.0)	<0.001	1325.5(725.1,1926.0)	<0.001	1597.9 (422.1, 2773.7)	0.008
Thrombolysis	697.2(334.7, 1059.7)	<0.001	854.1(553.4, 1154.8)	<0.001	501.7(−8.4, 1011.7)	0.054	–	–
Thrombectomy	2401.5 (−389.0, 5191.9)	0.092	3468.8(1936.9, 5000.6)	<0.001	7183.2 (5830.6, 8535.7)	<0.001	10397.1 (9231.1, 11563.1)	<0.001

*CI, confidence interval; DM, diabetes mellitus; CHD, coronary heart disease; AF, atrial fibrillation; ICU, intensive care unit; DVT, deep venous thrombosis*.

## Discussion

Regional differences in stroke have been widely reported worldwide in terms of outcomes and medical care (19–23) However, regional differences in hospital costs of AIS have rarely been reported, especially in China. In this study, we presented the components of hospital costs in different regions, explored the relationships among them and identified the determinants of total hospital costs in each region. These findings will provide evidences for strategy making to increase medical efficiency and reduce hospital costs.

Each study has different grouping methods, which will influence some provinces' affiliations. Generally, the annual incidence of stroke is 303.7–365.2 per 0.1 million in the northeastern area, 232.6–338.9 per 0.1 million in the eastern area, 319.4–326.1 per 0.1 million in the central area, and 83.9–316.2 per 0.1 million in the western area (3, 24). To better present the variations in hospital costs of AIS of each region from temporal aspect in China, we collected studies involving hospital costs of AIS conducted in China in the past 20 years and present them in Supplementary Table 1 (4–12). Compared with the past, hospital costs in each region have all increased, which is mainly caused by the development of medical technology and an increase in the economic level in China, and the hospital costs in China are much lower than those of developed counties (European: 3,214–6,845 dollars; the United States: 4,408–16,200 dollars; Japan: 6,887–8,662 dollars) (25–29). Different regions all show similar constitutive characteristics of hospital costs, which reflects the value feature of medicine care: Western medicine costs constitute a large proportion, and the value of medical personnel costs less in China, which is opposite to developed countries (27, 30). Traditional medicine costs takes the smallest proportion of the total cost in our study. A study from Beijing Public Health Information Center presented that the median costs of Chinese herb medicine was 0 dollar and the median cost of traditional Chinese patent medicine was 37 dollars, which was basically in line with our results (5). Another noteworthy point was insurance type, a study including 158,781 patients in Beijing reported that 58.7% of patients had medical insurance, 19.1% had new rural cooperative medical services, 7.6% were self-pay, and 14.5% paid by other payment types (5). From these data, we speculated that public insurance was the main insurance type. In this study, the thrombolysis rate was 4.6% which was in line with previous studies of 1–18% in China, and it was relatively low compared to Western developed counties (31, 32). Admission delay, high costs for patients, and high concern over bleeding risk among patients from families and doctors may contribute to a low thrombolysis rate (32). Furthermore, some socioeconomic effects such as education, level of income, place of residence, and means of transport to the hospital, can have effects on pre-hospital delay, which also contribute to a low thrombolysis rate (33). Although the western area had the highest thrombectomy rate, it is difficult to draw any robust conclusion on thrombectomy in consideration of the small number of thrombectomy in this study. For the effects of regions on hospital costs, subgroup analyses present more details. Regions have different influences on total costs among different stroke severity groups. Patients from areas with higher per capita disposable income tended to have higher total costs when they had non-mild stroke, especially moderate stroke severity. Whether patients underwent thrombolysis or not, patients from areas with higher per capita disposable income tended to have higher hospital costs, and this trend was more obvious when thrombolysis is involved, which will be costly. These differences may result from economic factor that people from areas with higher per capita disposable income can afford better medicine sources.

Generally, patients from developed regions have better access to good medical care and can afford it, and their hospital costs tend to be higher, which was in line with the results of the multivariate analysis in this study. Per capita disposable income can reflect each region's development level to an extent. The eastern area and the northeastern area, the two areas with higher per capita disposable income, have lower actual hospital costs compared with other two areas, which is in line with previous findings that patients with low socioeconomic status have higher hospital costs (34, 35). A longer LOS partially contributed to this cost difference. As previous studies suggest, patients with low socioeconomic status tend to have longer LOS due to poor access to primary care and rehabilitation centers, while developed regions could provide better primary care and have more rehabilitation centers (34, 35). Poor access to primary care can also lead to delayed diagnosis and more severe conditions, which further contribute to high hospital costs. And this is more common in developing regions. Another potential factor is medical policy. With the spread of diagnostic-related group pricing and payment policy and other related medical policy reforms in China, whose main targets were reducing the proportion of medical costs and increasing the proportion of labor costs to highlight the value of medical staff and reduce the overall health care expenditures of the nation, these policy reforms are executed more deeply in developed regions, and the results seem to be more obvious (36).

The determinants of hospital costs in each region varied extensively, which provides potential aspects to improve medical efficiency according to each region's condition. Some determinants were unalterable, such as age and sex, while others could be prevented or treated. Comorbidities contributed more to hospital costs in areas with higher per capita disposable income compared, which reflects stroke's complexity, considering that some patients were transported from other regions. LOS was an important factor for hospital costs in each region. The median LOS in the overall cohort was 12 days and has decreased compared to the past, ranging from 14 to 19 days (7–9). The median LOS in Western developed countries ranged from 8.8 to 11.8 days, indicating that there is still room to further reduce LOS in China, although patient outcomes and current medical conditions in China should be taken into consideration (25, 28, 37). Further studies are required to explore the determinants of LOS and identify the best LOS strategies for China. Another noteworthy common factor for hospital costs was hemorrhage, and the prevention and treatment of this factor should be given greater attention in the future.

The limitations of our study were also obvious. First, the study did not include the classifications of AIS, such as Trial of Org 10,172 in Acute Stroke Treatment (TOAST) classification, which have been proven to be related to hospital costs in young patients, and this limited the further exploration of hospital costs from the aspect of etiology (10). Second, some potential factors that we did not include in this study may also play important roles in hospital costs, such as patients' annual incomes, caregiving status. Third, patients from the western area accounted for a small proportion of the participants in this study, which resulted in insufficient evaluation of hospital costs in this area. Future studies are required to include more patients from the western area to better provide a profile of hospital costs in the western area. Fourth, it has been 4 years since the CASTOR study was completed, the popularization of intravenous thrombolysis and thrombectomy may have changed. Further studies should pay more attention to these treatments and explore the potential economic issues.

## Conclusion

In conclusion, this is the most extensive multicenter prospective study to explore regional differences in hospital costs of AIS in China in the last 10 years. Hospital costs in different regions show a similar proportion size order of components. Each region has different determinants of hospital costs, reflecting the current conditions of medicine and providing potential aspects for improvement to increase treatment levels and reduce hospital costs according to each region's situation.

## Data Availability Statement

The raw data supporting the conclusions of this article will be made available by the authors, without undue reservation.

## Ethics Statement

The study has been approved by the Ethics Committee of all participating hospitals. The patients/participants provided their written informed consent to participate in this study.

## List of Chinese Acute Ischemic Stroke Treatment Outcome Registry (CASTOR) Investigators

Xiaomu Wu, Jiangxi Provincial People's Hospital; Zhiyu Nie, Tongji Hospital; Xiangzhe Liu, The First Affiliated Hospital of Henan University of Chinese Medicine; Junfeng Shi, Nanshi Hospital of Nanyang City; Li Ding, The First People's Hospital of Yunnan Province; Dai Huang, Zhengzhou Central Hospital; Ning Wang, The First Affiliated Hospital of Fujian Medical University; Jingfen Zhang, Inner Mongolia Medical University; Ruiyou Guo, Hiser Hospital of Qingdao; Xuerong Qiu, Qiqihar First Hospital; Jun Wu, Peking University Shenzhen Hospital; Yan Liu, Guangzhou General Military Hospital of the Chinese People's Liberation Army; Lianbo Gao, The Fourth Affiliated Hospital of China Medical; Xingjuan Zhao, First People's Hospital of Zhengzhou; Yuhui Han, Gongyi People's Hospital; Xinling Meng, Autonomous Region Hospital of Traditional Chinese Medicine Affiliated to 17, Xinjiang Medical University; Xuhai Gong, Daqing Field General Hospital; Jie Han, The First Afiliated Hospital of Dalian Medical university; Yongbo Zhang, Beijing Friendship Hospital Affiliated to Capital Medical University; Jinzhao Wang, Yutian County Hospital of Tangshan City; Shuhong Ju, Liqun Hospital of Putuo District; Zhilin Jiang, 202 Hospital of the Chinese People's Liberation Army; Shuyan Zhang, The Fourth Affiliated Hospital of Harbin Medical University; Deyang Li, Affiliated Central Hospital of Tengzhou; Wenwei Yun, The Second People's Hospital of Changzhou City Affiliated to Nanjing Medical; Xueqiang Hu, The Third Affiliated Hospital of Sun Yat-sen University; Juming Yu, Affiliated Hospital of North Sichuan Medical College; Junyan Liu, The Third Hospital of Hebei Medical University; Zhiyun Wang, Tianjin First Central Hospital; Deqin Geng, The Affiliated Hospital of Xuzhou Medical University; Yukai Wang, The First Hospital of Foshan; Peiyuan Lv, Hebei General Hospital; Danhong Wu, Shanghai Ninth People's Hospital; Yangtai Guan, Renji Hospital; Cuilan Wang, Qilu Hospital of Shangdong University; Qingchun Gao, The Second Affiliated Hospital; Xuejun Zhang, Second Hospital Affiliated to Xinjiang Medical University; Hongmei Liu, Xiyuan Hospital of China Academy of Chinese Medicine Sciences; Tao Gong, Beijing Hospital; Shujuan Tian, The 260th Hospital of the Chinese People's Liberation Army; Shuijiang Song, The Second Affiliated Hospital of Zhejiang University School of Medicine; Shaoshi Wang, Branch of Shanghai General Hospital; Haiqing Song, Capital Medical University Xuanwu Hospital; Zuneng Lu, Wuhan University Renmin Hospital; Xiaoxiang Peng, The Third People's Hospital of Hubei; Qi Fang, The First Affiliated Hospital of Soochow University; Wendan Tao, West China Hospital; Shilie Wang, Jiujiang No.1 People's Hospital; Ping Zhang, The First Hospital Affiliated to Xinxiang Medical University; Xiaojie Wang, Fushun Third Hospital; Qiang Zhang, Fushun Central Hospital; Weishu Xue, Harbin No.4 Hospital; Liping Wei, Affiliated Luoyang Central Hospital of Zhengzhou University; Weiliang Luo, Huizhou Central People's Hospital; Yuling Jin: The First Affiliated Hospital of Jiamusi University; Juan Feng, Shengjing Hospital of China Medical University; Xinyan Wu, The Third Affiliated Hospital of Xinxiang Medical University; Yongzhong Lin, The Second Affiliated Hospital of Dalian Medical University; Hongbing Nie, The Affiliated Hospital of Jiujiang University; Wei Liu, Donggang Central Hospital; HuiCao, Brain Hospital Affiliated to Nanjing Medical University; Qi Tan, Affiliated Donghua Hospital of Sun Yat-sen University; Yude Zhang, The First Affiliated Hospital of Henan University of Science and Technology; Youqing Deng, First Hospital of Nanchang; Guangxian Nan, China-Japan Union Hospital of Jilin University; Lishu Wan, The First Hospital of Dandong; Xiaoping Yin, The Second Affiliated Hospital of Nanchang University; Wenyan Zhuo, Zhuhai People's Hospital; Bingzhen Cao, General Hospital of Jinan Military Command.

## Author Contributions

YL, HJ, and YH: conceptualization and methodology. YL and ZS: data curation. HJ, WeiS, RL, FL, JS, LT, GL, HC, GZ, LZ, XS, JQ, and YW: investigation. WeipS and YH: supervision. YL: writing—original draft. HJ and YH: writing—review and editing. All authors have read and agreed to the published version of the manuscript.

## Funding

This work was supported by the National Natural Science Foundation of China (No. 81400944) and Peking University Medicine Seed Fund for Interdisciplinary Research (No. BMU2018MX020). The CASTOR study was funded by Techpool Bio-Pharma Co., Ltd. All funders were not involved in the study design, collection, analysis, interpretation of data, the writing of this article, or the decision to submit it for publication.

## Conflict of Interest

The authors declare that the research was conducted in the absence of any commercial or financial relationships that could be construed as a potential conflict of interest.

## Publisher's Note

All claims expressed in this article are solely those of the authors and do not necessarily represent those of their affiliated organizations, or those of the publisher, the editors and the reviewers. Any product that may be evaluated in this article, or claim that may be made by its manufacturer, is not guaranteed or endorsed by the publisher.
