# Supplementary material for: Regional Differences in Hospital Costs of Acute Ischemic Stroke in China: Analysis of Data From the Chinese Acute Ischemic Stroke Treatment Outcome Registry
Source: Front Public Health. 2021 Dec 10;9:783242. doi: 10.3389/fpubh.2021.783242 (PMC8702643; doi:10.3389/fpubh.2021.783242)
Supplement: Supplementary file 1 [file Table_1.DOCX]

**Table S1.** Comparison of hospitalization costs of ischemic stroke studies in China

|  | **Current study** | **Liu et al.** | **Zhang et al.** | **Huang et al.** | **Kong et al.** | **Li and Luo** | **Huo et al.** | **Wei et al.** | **Ma et al.** | **Tu et al.** |
| --- | --- | --- | --- | --- | --- | --- | --- | --- | --- | --- |
| Study type | Prospective | Cross-sectional | Cross-sectional | Retrospective | Retrospective | Retrospective | Retrospective | Prospective | Retrospective | Retrospective |
| Study start time | 2015 | 2015 | 2006 | 2012 | 2012 | 2014 | 2012 | 2006 | 2008 | 1997 |
| Recruitment duration/follow-up time | 2 years | 1 year | 7 years | 4 years | 3 years | 1 year | 1 year | 5 months | 1 year | 2 years |
| Regional extent | Multiprovincial | Multiprovincial | Guangzhou (the eastern area) | Jiangxi (the central area) | Beijing (the eastern area) | Guangdong (the eastern area) | Beijing (the eastern area) | Multiprovincial | Sichuan (the western area) | Beijing (the eastern area) |
| Sample size | 8547 | 3143 | 86126 | 961 | 158781 | 824 | 83121 | 3852 | 557 | 545 |
| Male, n (%) | 5604 (65.6%) | 1830 (58.2%) | 46335 (53.8%) | 733 (76.3%) | 100120 (63.1%) | 526 (63.8%) | 50652 (60.9%) | None | 328 (58.9%) | 392 (72.1%) |
| Age, year | 64.2±12.0 | 63.4±14.5 | 72.5±10.9 | 42.1±5.7 | 67.7±12.4 | 64.7±12 | 67.4±12.7 | None | 66.5±11.1 | 65^b^ |
| Medical history |  |  |  |  |  |  |  |  |  |  |
| Stroke, n (%) | 2044 (23.9%) | None | None | None | None | None | None | None | None | 246 (45.1%) |
| Hypertension, n (%) | 5497 (64.3%) | None | 40221 (46.7%) | 499 (51.9%) | 113852 (71.7%) | 564 (68.4%) | 57617 (69.3%) | None | 355 (63.7%) | 334 (61.3%) |
| DM, n (%) | 2167 (25.4%) | None | 16278 (18.9%) | 185 (19.3%) | 55823 (35.2%) | 217 (26.3%) | 25264 (30.4%) | None | 90 (16.2%) | 131 (24.0%) |
| CHD, n (%) | 1182 (13.8%) | None | 12404 (14.4%) | None | None | None | None | None | 113 (20.2%) | 102 (18.7%) |
| AF, n (%) | 381 (4.5%) | None | None | 279 (29.0%) | None | 73 (8.86%) | None | None | None | None |
| Stroke severity | NIHSS:  0–5: 63.7%  6–10: 22.6%  >10: 13.7% | None | None | NIHSS (Mean±SD): 7.36±5.32 | None | NIHSS:  ≤8: 73.1%  9–15: 17.1%  ≥16: 9.3% | None | None | NIHSS:  0–6: 72.2%  7–15: 21.0%  16–38: 6.8% | SSS:  9–19: 2.0%  20–29: 4.6%  30–39: 12.5%  40–48: 80.9% |
| Complications |  |  |  |  |  |  |  |  |  |  |
| Infections | 7.1% | 8.7% | None | None | 13.8% | 20.91% | None | None | None | 9.7% |
| DVT | 0.1% | 0.8% | None | None | None | None | None | None | None | None |
| Hemorrhage | 1.9% | None | None | None | None | None | None | None | None | None |
| Length of stay, days | 12.0 (9.0–15.0) | 14.5±11.6 | 25.1±52.7 | None | 13.5 (9.9–18.1) | 11.5±9.3 | 14 (9–19) | 19^a^ | 16.0 (8.5–23.5) | 32^b^ |
| Total costs, dollar | 2248.0 (1507.3–3339.2) ^b^ | 2662.5 (1126.8–5835.9) ^b^ | 2818.8±4082.7^a^ | 5958.4^a^ | 2112 (1436–3147) ^b^ | 2393.7±2356.4 ^a^ | 2706.2±2931.9 ^a^ | 1527^a^ | 812.6 (483.4–1167.1) ^b^ | 1104.5^c^ |

a, Mean value±standard deviation; b, median value (interquartile ranges); c, geometric mean; None, unavailable; DM, diabetes mellitus; CHD, coronary heart disease; AF, atrial fibrillation; DVT, deep venous thrombosis; NIHSS, the National Institutes of Health Stroke Scale; SD; standard deviation; SSS, the modified scale of the Scandinavian stroke scale.
